# TBK1 has a new Akt

**DOI:** 10.1016/j.jbc.2021.101244

**Published:** 2021-09-24

**Authors:** Leslie M. Shaw

**Affiliations:** Department of Molecular, Cell & Cancer Biology, University of Massachusetts Chan Medical School, Worcester, Massachusetts, USA

**Keywords:** EGF, epidermal growth factor, MEFs, mouse embryonic fibroblasts, mTOR, mechanistic target of rapamycin, mTORC1, mTOR complex 1, mTORC2, mTOR complex 2, TBK1, TANK-binding kinase 1

## Abstract

TANK-binding kinase 1 (TBK1) is a noncanonical IκB kinase that plays an essential role in the innate immune response to foreign pathogens. Recent studies have highlighted additional roles for TBK1 in the regulation of metabolism, although the mechanisms of this regulation have not been well characterized. In a recent issue, Tooley *et al.* demonstrated that TBK1-dependent activation of downstream kinase Akt is mediated *via* mechanistic target of rapamycin complex 2. This novel action of TBK1 reveals a key role for this kinase in the regulation of cellular metabolism and growth by diverse environmental inputs.

TANK-binding kinase 1 (TBK1), a serine/threonine kinase that belongs to the noncanonical IκB kinase family, plays an essential role in the innate immune response to viral and bacterial pathogens by regulating the type I interferon–mediated T cell response (1). Although TBK1 has been most widely studied in this context, more recent investigations using tissue-specific KO mice and drugs that inhibit kinase activity have revealed novel roles for this kinase in nonimmune cells, particularly at the intersection of immunity and metabolism. For example, TBK1 expression and activity are induced in adipose tissue in obesity by elevated expression of proinflammatory cytokines such as tumor necrosis factor α (2). TBK1 contributes to obesity by repressing energy expenditure and increasing anabolic functions as determined from analysis of mice with conditional adipose cell KO of TBK1 (3). TBK1 has also been reported to promote activation of Akt, a central kinase involved in metabolic regulation (4). However, the mechanism by which TBK1 regulates Akt has remained unclear.

Akt is an essential regulator of glucose metabolism and plays an important role in controlling cellular glucose uptake and utilization through both positive and negative regulatory actions (4). Phosphorylation of Akt on T308 in its activation loop stimulates kinase activity, and phosphorylation on S473 further enhances activity and determines substrate specificity (4). Although it had been previously reported that TBK1 can directly phosphorylate Akt at S473 and T308 in *in vitro* kinase assays, the ability of TBK1 to mediate these phosphorylation events under physiological conditions was not known (5). In a recent study, Tooley *et al*. (6) contributed to the mechanistic understanding of TBK1 function in metabolic regulation by demonstrating a role for TBK1 in mechanistic target of rapamycin (mTOR) complex 2 (mTORC2) activation and subsequent phosphorylation of Akt.

To investigate how TBK1 regulates Akt activation, mouse embryonic fibroblasts (MEFs) were stimulated with epidermal growth factor (EGF) and evaluated for Akt-S473 and Akt-T308 phosphorylation (6). The intensity and duration of Akt phosphorylation at both sites was diminished significantly, both in the absence of TBK1 and in the presence of the TBK1 inhibitor amlexanox. Restoration of endogenous levels of TBK1, but not kinase-dead TBK1, rescued EGF-stimulated Akt-S473 phosphorylation. The stimulation of Akt-S473 phosphorylation by EGF, as well as by other growth factors and the hormone insulin, was found to be dependent upon mTOR activity. Together, these results validate the ability of TBK1 to regulate Akt-S473 phosphorylation and show that in response to normal growth regulatory signaling, this regulation is mediated through mTOR kinase.

The kinase mTOR is the core catalytic kinase of two multisubunit complexes, mTOR complex 1 (mTORC1) and mTORC2, which are distinguished by the scaffolding proteins Raptor and Rictor, respectively (7). mTORC1 is regulated by the combination of growth factor/hormone signaling and nutrient availability to drive anabolic metabolism. mTORC2, on the other hand, is regulated by growth factor/hormone signaling to activate Akt. Together, mTORC1 and mTORC2 are key signaling nodes in the regulation of cell growth and proliferation, and dysregulation of these signaling pathways contributes to metabolic disease and cancer. In previous investigations, the authors had demonstrated that phosphorylation of mTOR on S2159 by TBK1 enhanced mTORC1 activation and downstream signaling to promote cell growth and proliferation (8). To investigate if TBK1 acts upstream of mTORC2 to regulate Akt-S473 phosphorylation through a similar mechanism, MEFs derived from mice with an alanine knock-in at S2159 (*Mtor*^*A/A*^) were stimulated with EGF. A marked reduction of Akt-S473 phosphorylation was observed in *Mtor*^*A/A*^ MEFs compared with WT MEFs (*Mtor*^*+/+*^). Using immunoprecipitation of Rictor to isolate the mTORC2 complex, TBK1 was observed to interact with mTORC2 and directly phosphorylate mTOR-S2159 to activate mTORC2 intrinsic kinase activity toward Akt-S473. TBK1 activity is increased by phosphorylation of S172 in its activation loop in response to pathogens in the innate immunity pathway. In contrast, Tooley *et al.* (6) found that EGF stimulation did not enhance S172 phosphorylation, supporting that it is the basal activity of TBK1 that is important for mTORC2 signaling downstream of growth factors. However, when RAW264.7 macrophages and primary bone marrow–derived macrophages were stimulated with the dsRNA mimetic poly(I:C), which induces TBK1-S172 phosphorylation, TBK1 and mTOR-S2159 were also found to be required for mTORC2-dependent phosphorylation of Akt-S473. Finally, the physiological regulation of mTORC2 activity by TBK1 was assessed by injection of *Mtor*^*A/A*^ and *Mtor*^*+/+*^ mice with poly(I:C). Spleen tissue isolated from *Mtor*^*A/A*^ mice showed diminished Akt-S473 phosphorylation. Therefore, the authors conclude that under both basal and activated states, the activation of Akt by TBK1 is mediated through mTORC2 (Fig. 1) (6).

**Figure 1:**
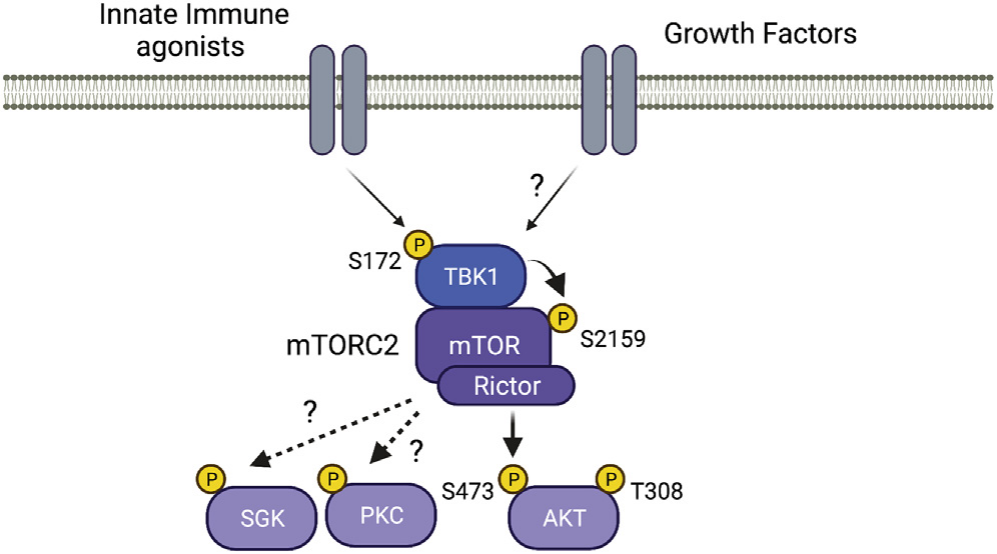
**TBK1 promotes AKT activation through mTORC2.** TBK1 interacts with and phosphorylates mTORC2 on S2159 of mTOR in response to either growth factor stimulation or innate immune agonists to promote AKT activation. Created using BioRender.com. mTORC2, mTOR complex 2; SGK, serum/glucocorticoid-regulated kinase; TBK1, TANK-binding kinase 1.

TBK1 regulation of mTORC2-dependent phosphorylation of Akt shown in this study adds to the growing role of TBK1 as a signaling node in the regulation of cellular metabolism and growth by diverse environmental inputs. In response to foreign pathogens or inflammatory cytokines that stimulate TBK1 activation, or growth factor/hormone signaling that requires basal TBK1 activity, mTORC2 is activated to promote Akt-S473 phosphorylation and its downstream functions. Given that TBK1 expression and activity are enhanced in metabolic diseases and cancer, and the important role that Akt plays in these pathological conditions, identifying TBK1 as an upstream regulator of Akt reveals a potential novel approach to disrupt this signaling axis for therapeutic benefit (4, 9). In this regard, drugs such as amlexanox and other compounds are under investigation for their potential clinical use (10). Of note, the study by Tooley *et al.* (6) only examined the TBK1-dependent phosphorylation of Akt-S473 by mTORC2; mTORC2 also has additional substrates, including serum/glucocorticoid-regulated kinase and members of the PKC family (Fig. 1) (4). These kinases regulate unique cellular functions, such as regulation of the actin cytoskeleton. It will be important to determine if TBK1 regulates the activation of these kinases through mTORC2 as well, to understand the full impact of inhibiting TBK1 function therapeutically.

The mechanism by which TBK1 regulates mTORC2 function has not been established. Although the kinase activity of TBK1 is required for Akt-S473 phosphorylation, neither phosphorylation of S172 in the activation loop of TBK1 nor phosphorylation of mTOR-S2159 was increased by growth factor stimulation in this study. Phosphorylation of S172 stabilizes the active confirmation of TBK1 and it is possible that additional uncharacterized phosphorylation sites could serve a similar function. Alternatively, the interaction of TBK1 with mTORC2 could impact TBK1 conformation, or multimerization, to enhance activity. Intracellular localization of mTORC2 could also be determined by TBK1 interaction, which could affect substrate availability. As little is known about the upstream regulation of mTORC2, the next acts should be elucidating further the mechanism of its activation by TBK1 to reveal novel approaches for targeting the mTORC2-Akt signaling pathway.

## Conflict of interest

The author declares that she has no conflicts of interest with the contents of this article.
